# Abnormal uterine bleeding and its associated factors among reproductive-age women who visit the gynecology ward in Dilla University General Hospital, Southern Ethiopia, 2022

**DOI:** 10.1186/s12905-024-03128-6

**Published:** 2024-05-09

**Authors:** Mesfin Abebe, Getnet Melaku, Habtamu Endashaw Hareru, Tsion Mulat Tebeje

**Affiliations:** 1 Department of Midwifery, College of Medicine & Health Sciences, Dilla University, Dilla, Ethiopia; 2 School of Public Health, College of Medicine & Health Sciences, Dilla University, Dilla, Ethiopia

**Keywords:** Magnitude, Abnormal uterine bleeding, Associated factors, Reproductive age women

## Abstract

**Background:**

Abnormal uterine bleeding, a frequent gynecological problem among women of reproductive age, significantly affects their health and quality of life. Despite its problem, research on its extent and contributing factors in Ethiopia is scarce. Hence, this study is designed to determine the magnitude and factors associated with abnormal uterine bleeding among women visiting Dilla University General Hospital, Dilla, Ethiopia.

**Methods:**

A cross-sectional study design was conducted with 380 women of reproductive age at Dilla University General Hospital. A systematic sampling method was employed to select the participants for the study. A structured interview administered questionnaire and checklist were used to collect the data. Stata V.14 software was used for cleaning, coding, ensuring completeness and accuracy, and further analysis. Bivariate and multivariable logistic regression analyses were used. Finally, the variables that have a *p*-value of < 0.05 were considered statistically significant.

**Results:**

In this study, the magnitude of abnormal uterine bleeding was 24.21% (95% CI, 20.14–28.79). History of sexually transmitted disease [AOR = 1.44, 95% CI: (1.33, 4.75)], history of anemia [AOR = 3.92, 95% CI: (1.20, 12.74)]., history of alcohol consumption [AOR = 2.49, 95% CI: (1.22, 5.06)], and perceived stress level [AOR = 1.30, 95% CI: (1.15, 1.69)] were found to be significantly associated with abnormal uterine bleeding.

**Conclusions:**

The magnitude of abnormal uterine bleeding was 24.2% in the study setting. Factors such as a history of sexually transmitted disease, anemia, alcohol consumption, and perceived stress level were identified as significant risk factors for abnormal uterine bleeding. Addressing these factors is crucial for management. Further research and interventions targeting these risks are needed to enhance health outcomes. The study provides valuable insights for future interventions.

**Supplementary Information:**

The online version contains supplementary material available at 10.1186/s12905-024-03128-6.

## Introduction

Abnormal uterine bleeding (AUB) is defined as any menstrual bleeding from the uterus that is either abnormal in volume, regularity, or timing, or that is non-menstrual [1]. It is a common gynecologic concern in women of reproductive age [2, 3]. The magnitude of AUB varies across populations, ranging from 10–30% [4]. It reduces quality of life and is linked to financial setbacks, decreased efficiency, worsening health, and greater healthcare costs [5]. In the United States, menorrhagia accounts for more than 18% of all gynecology outpatient visits [6]. Every year, over 800,000 women seek treatment for AUB in the United Kingdom. Along with the direct impact on the woman and her family, there are significant economic and healthcare costs [7]. AUB is a major cause of gynecological morbidity, affecting one out of every five women at some point during their reproductive period [8]. It is a medically and socially debilitating disorder. Furthermore, it is the most common cause of iron deficiency in the developed world, as well as chronic illness in the developing world [9].

From puberty to menopause, abnormal uterine bleeding is a common complaint among women. It hurts women’s health by causing anemia and lowering their quality of life. One of the most common reasons reproductive-aged women seek medical help is for AUB [6, 10]. The pattern and factors that cause AUB differ depending on the woman’s age and reproductive health [8]. AUB accounts for roughly one-third of all visits to gynecologists among premenopausal women and more than 70% of office visits among perimenopausal and postmenopausal [11]. The estimated annual direct and indirect costs of abnormal bleeding are $1 billion and $12 billion, respectively [1]. Abnormal bleeding is also a common reason for women being referred to gynecologists, and it is a reason for up to 25% of all gynecologic surgeries [6].

According to studies conducted in Iran, India, Ethiopia, and Korea, the prevalence of abnormal uterine bleeding was 35.8%, 20.48%, 34.1%, and 14.3% respectively [8, 12–14]. Pieces of evidence revealed that age, multiparty, body mass index, perceived stress, history of abortion, history of uterine fibroid, history of sexually transmitted infection, and history of IUCD were all contributing factors to abnormal uterine bleeding [3, 8, 12–14].

Abnormal uterine bleeding is a common condition that can result in significant morbidity, and women with abnormal uterine bleeding have a significantly lower quality of life than the general population [15]. Due to the scarcity of information on abnormal uterine bleeding and associated factors among women of reproductive age in Ethiopia and the study area, this current study is very important. By addressing this important public health issue, we aim to improve women’s health outcomes and enhance healthcare services in the region. This study aims to investigate the magnitude and factors associated with abnormal uterine bleeding among women attending Dilla University General Hospital, Southern Ethiopia.

## Methods and materials

### Study area, design, and period

A facility-based cross-sectional study was conducted at Dilla University General Hospital, Dilla Town. Dilla University General Hospital is the only public hospital in town. Dilla University General Hospital serves as a referral hospital for all districts in the Gedeo zone, as well as some districts in Oromia and Sidama regions. The hospital in various wards provides preventive, curative, and rehabilitative services to a catchment population of 2 million people. The study was conducted from June 20 -August 20, 2022.

### Population

All reproductive-age women (15–49 years) who visited the gynecology outpatient department at Dilla University General Hospital were the source population. Selected reproductive-age women (15–49 years) who visited the gynecology outpatient department at Dilla University General Hospital during the study period in 2022 were the study population.

### Eligibility criteria

Women in the reproductive age group of 15–49 years were included in the study. Those who were seriously ill, pregnant at the time of the study, who had undergone a hysterectomy or other surgical procedures that could affect their menstrual bleeding patterns, with a known history of bleeding disorders or coagulopathies, and unable to communicate during the data collection period were excluded.

### Sample size determination

The sample size was calculated by using a single proportion formula by considering the assumptions: *P* = 34.1% the magnitude of AUB done in Jimma town [13], Oromia Region, Southwest Ethiopia. D = 5% the margin of error, Zα/2 = 1.96 at 95% confidence of certainty. Thus, n= ((Zα/2)2 * p (1 − p))/d2 = 345, considering 10% non-response rate = 35 and the final sample size was 380.

### Sampling techniques and procedure

Dilla University General Hospital was selected. The total number of reproductive-age women visiting the Gynecology outpatient department in the previous year was obtained from the hospital’s registration log book. Then K^th^ value was calculated by taking the previous year’s three-month period reports of the total number of reproductive-age women who visited the Gynecology outpatient department which was 750/380 ≈ 2. A systematic random sampling technique was used to choose our study participants. The first participant was selected randomly through a lottery method, and then every K^th^ interval was chosen to identify subsequent participants for the study.

### Study variables

#### Dependent variable

Abnormal uterine bleeding (Yes or No).

#### Independent variables

**Sociodemographic factors** (age, marital status, residence, educational status, monthly income, occupational status) **Reproductive health and clinically diagnosed related factors** (parity, history of abortion, history of STI, history of IUCD, history of hormonal contraceptive, uterine cancer, bleeding disorder, thyroid disorder, anemia) **Lifestyle behavior** (drinking alcohol, smoking, BMI, stress).

### Operational definition

Abnormal Uterine Bleeding (AUB) can be characterized by any of the following conditions: heavy menstrual bleeding, metrorrhagia, polymenorrhea, Oligomenorrhea, amenorrhea, or bleeding between menstrual cycles.

The **Perceived Stress Scale** was used to assess stress levels (PSS). PSS is a 10-item multiple-choice self-report psychological instrument for measuring stress perception. Each response was assigned a score ranging from 0 to 4. PSS is calculated by adding the totals of all scale items [16, 17].

### Data collection tools and procedures

The data were collected using a questionnaire that was developed after reviewing various pieces of literature. A structured interview administered questionnaire and checklist were used to collect the data. The questionnaire includes socio-demographic information, menstrual-related questions, lifestyle and behavioral questions, medical history questions, and anthropometric measurements (height and weight). Data collectors and supervisors were trained on data collection tools, interview techniques, information confidentiality, and the study’s objective and relevance. The Kobo Collect version 3.1 application was installed on the data collector’s Android phone, and the blank form was downloaded from the Kobo toolbox server.

### Data quality control

To maintain data quality, the questionnaire was translated into the local language by a language expert. Data collectors and supervisors received two days of training to become familiarized with all types of data, tools, data collection methods, and study objectives. The questionnaires were pre-tested three weeks before the actual data collection. To ensure data quality, supervisors checked completed questionnaires for key content before uploading them from the Android mobile phone to the Kobo toolbox server. All data were collected on-site using Android mobile devices and uploaded to the Kobo server weekly using Kobo collect version 3.1. Regularly, the principal investigator checked the sent files from each data collector for consistency and completeness. Finally, to ensure data quality, proper data coding and categorization were performed.

### Data processing and analysis

The data from the Kobo server was downloaded as an Excel file and exported to the Stata V.14 software for cleaning, coding, ensuring completeness and accuracy, and further analysis. Both bivariate and multivariable logistic regression analyses were used to assess the association between each independent variable and the outcome variable. Variables with a 95% confidence interval and a *P*-value of less than 0.25 in the bivariate analysis were included in the multivariable logistic regression analysis to control for all potential confounding variables. The Hosmer-Lemeshow goodness fitness test was performed to assess model fitness. The variance inflation factor (VIF) was used to assess multicollinearity amongst independent variables. Finally, adjusted odds ratios with 95% confidence intervals were calculated, and *P*-values less than 0.05 were considered statistically significant. Finally, data was presented in the form of tables, graphs, and text.

## Results

### Sociodemographic and economic characteristics of study participants

In this study, 380 study participants were interviewed, with a 100% response rate. The mean and standard deviation of the study participants’ age was (28 ± 6.5) years. In terms of religion, about 236 (62.11%) of the women were Protestant, while 116(30.53%) women were Orthodox. Three hundred nine women (81.32%) of those respondents lived in urban areas. In terms of marital status, 250 (65.78%) of respondents were married.

In terms of the educational and occupational status of study participants, 92 (24.21%) had no formal education, and 200 (52.63%) were housewives. The median and interquartile range of monthly income was 100 Ethiopia Birr (IQR: 500, 2000). 193 (50.79%) of study participants earned less than 1000 (ETB) per month (Table 1**).**

**Table 1:** Socio-demographic and economic characteristics of study participants in Dilla University General Hospital, Southern Ethiopia, 2022(*N* = 380)

Variable	Frequency	Percentage (%)
**Age (in years)**		
15–19	29	7.63
20–24 25–29 30–34	95 108 68	25.00 28.42 17.89
≥35	80	21.05
**Religion**		
Orthodox	116	30.53
Muslim	28	7.37
Protestant	236	62.11
**Ethnicity**		
Gedeo	100	26.31
Guraghe	150	39.47
Oromo Siliti	44 50	11.57 13.15
Others®	36	9.47
**Residence**		
Urban	309	81.32
Rural	71	18.68
**Marital status**		
Single	100	26.31
Married	250	65.78
Widowed±	30	7.89
**Educational status**		
No formal education	92	24.21
Primary school	112	29.47
Secondary school	115	30.26
College and above	61	16.05
**Occupational status**		
Housewife	200	52.63
Farmer	29	7.63
Merchant	73	19.21
Government employee	66	17.36
Others**	12	3.15
**Household monthly income (ETB)**		
≤1000	193	50.79
1100/2000	113	29. 74
≥2100	74	19.47

***Key note***: ® Amhara, Tigre, ± separated, divorce **daily labour, self-employed, student

### Reproductive health and clinically diagnosed related characteristics of study participants

About 275 (72.37%) of the study’s participants were multiparous. Three hundred forty-two (90%) of women had no history of abortion. Out of the participants, 318(83.68%) women had a history of hormonal contraceptive usage. Seventeen (4.47%) of the respondents had a history of STI and anemia. 13(3.42%) of respondents had a previous history of uterine cancer and 27(7.11%) of participants had a history of bleeding disorder (Table 2**).**

**Table 2:** Reproductive health and clinical diagnosis-related characteristics of study participants in Dilla University General Hospital, Southern Ethiopia, 2022(*N* = 380)

Variable	Frequency	Percentage (%)
**Number of parity**		
Multiparous	275	72.37
Nulliparous	105	27.63
**History of abortion**		
Yes	38	10.00
No	342	90.00
**History of hormonal contraceptive used**		
Yes	318	83.68
No	62	16.32
**History of sexually transmitted infection**		
Yes	17	4.47
No	363	95.53
**History of previously diagnosed anemia**		
Yes	17	4.47
No	363	95.53
**History of previously diagnosed uterine cancer**		
Yes	13	3.42
No	367	96.58
**History of previously diagnosed bleeding disorder**		
Yes	27	7.11
No	353	92.89

### Lifestyle behaviour-related characteristics of study participants

In terms of smoking and drinking alcohol, about 35(9.21%) and 39(10.26%) of women smoked and drank alcohol at least once a month, respectively. Around 281 (73.95%) of the participants had a body mass index between 18.5 and 24.9 kg/m^2^. Out of the study participants, 136(35.79%) women had high levels of stress level (Table 3).

**Table 3:** Lifestyle behavior-related characteristics of study participants in Dilla University General Hospital, Southern Ethiopia, 2022(*N* = 380)

Variable	Frequency	Percentage (%)
**History of cigarette smoking**		
Yes	35	9.21
No	345	90.79
**History of alcohol consumption**		
Yes	39	10.26
No	341	89.74
**Body mass index**		
<18.5 18.5/24.9	45 281	11.84 73.95
≥25	54	14.21
**Perceived stress level**		
Low level	244	64.21
High level	136	35.79

### Magnitude of abnormal uterine bleeding

**Fig. 1:**
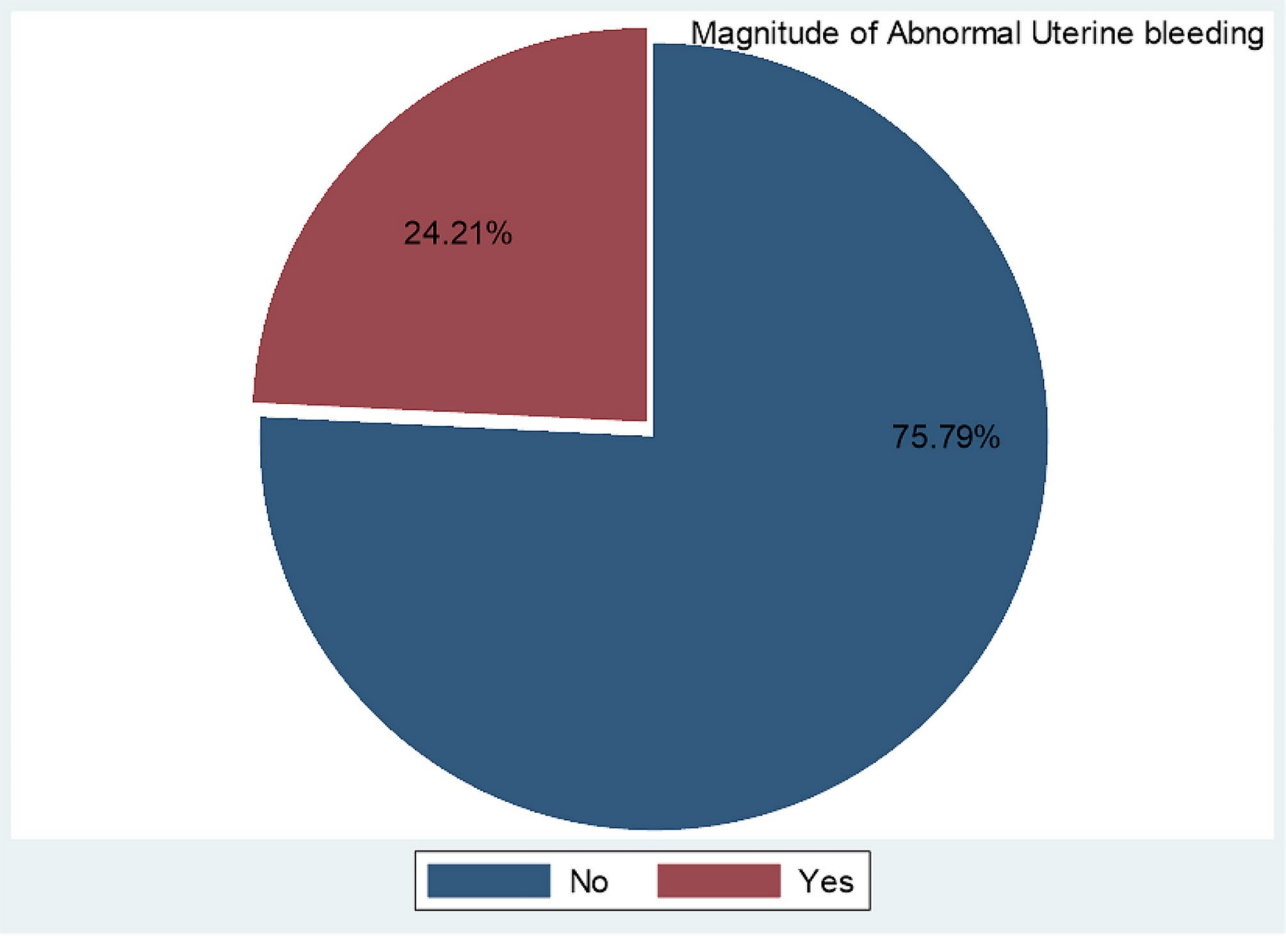
Magnitude of abnormal uterine bleeding among study participants in Dilla University General Hospital, Southern Ethiopia, 2022(*N* = 380)

In this study, the magnitude of abnormal uterine bleeding among study participants was 24.21% (95% CI, 20.14–28.79) (Fig. 1). Among the participants with AUB, the magnitude of heavy periods, metrorrhagia, polymenorrhea, oligomenorrhea, amenorrhea, and inter-menstrual bleeding in women of reproductive age was 27 (29.35%), 22 (23.92%), 15 (16.30%), 12 (13.04%), 7 (7.61%), and 9 (9.78%), respectively( Table 4).

**Table 4:** Pattern of AUB among reproductive-age women in Dilla University General Hospital(*N* = 92)

Pattern of AUB	Frequency	Percentage (%)
**Heavy period**	27	29.35
**Metrorrhagia**	22	23.92
**Polymenorrhea**	15	16.30
**Oligomenorrhea**	12	13.04
**Amenorrhea**	7	7.61
**Intermenstrual bleeding**	9	9.78

### Factors associated with abnormal uterine bleeding

To identify the associated factors of abnormal uterine bleeding, variables with a *p*-value of less than 0.25 in bivariate analysis, significant associations in previous studies, and biological plausibility were considered for the multivariable model. In binary logistics regression analysis, residence, history of hormonal contraceptive used, history of abortion, history of STI, history of anemia, number of parity, history of cigarette smoking, history of alcohol consumption, and perceived stress level were found to be candidate variable for final multivariable logistics regression analysis model.

In the multivariable analysis history of STI, history of anemia, history of alcohol consumption, and perceived stress level were found to be significantly associated with abnormal uterine bleeding. The odds of abnormal uterine bleeding were 1.44 times higher in women who had a history of sexual transmission infection compared to women who hadn’t STI [AOR = 1.44, 95% CI: (1.33, 4.75)]. Women who had a history of anemia were 3.92 times more likely to have abnormal uterine bleeding than those who had no history of anemia [AOR = 3.92, 95% CI: (1.20, 12.74)].

Women who had a history of alcohol consumption were 2.49 times more likely to have abnormal uterine bleeding than women who had no history of alcohol consumption [AOR = 2.49, 95% CI: (1.22, 5.06)]. In comparison to women who had low perceived stress levels, those who have high perceived stress levels have a 1.30 times higher risk of having abnormal uterine bleeding [AOR = 1.30, 95% CI: (1.15, 1.69)] (Table 5).

**Table 5:** Bivariate and multivariable logistic regression analysis of factors associated with abnormal uterine bleeding among study participants in Dilla University General Hospital southern Ethiopia, 2022(*N* = 380)

Variables	Abnormal uterine bleeding		COR (95%CI)	AOR (95%CI)	*P*-value
	Yes	No			
**Residence**					
Urban	73(79.35%)	236(81.94%)	1	1	
Rural	19(20.65%)	52(50.81%)	1.18(0.65,2.12)	1.34(0.72,2.50)	0.34
**History of hormonal contraceptive used**					
Yes	79(85.87%)	239(82.99%)	1.24(0.64,2.41)	1.43(0.68,3.00)	0.33
No	13(14.13%)	49(17.01%)	1	1	
**History of abortion**					
Yes	12(13.04%)	26(9.03%)	1.51(0.72,3.13)	1.73(0.80,3.75)	0.16
No	80(86.96%)	262(90.97%)	1	1	
**History of STI**					
Yes	8(8.70%)	9(3.12%)	2.95(1.10,7.89)*	1.44(1.33,4.75)**	**0.04**
No	84(91.30%)	279(96.88%)	1	**1**	
**History of anemia**					
Yes	10(10.87%)	7(2.43%)	4.89(1.80,13.26)*	3.92(1.20,12.74)**	**0.02**
No	82(89.13%)	281(97.57%)	1	1	
**Number of parity**					
Multiparous	64(69.57%)	211(73.26%)	0.83(0.49,1.39)	0.68(0.38,1.23)	0.20
Nulliparous	28(30.43%)	77(26.74%)	1	**1**	
**History of cigarette smoking**					
Yes	14(15.22%)	21(7.29%)	2.28(1.10,4.69)*	1.71(0.77,3.80)	0.18
No	78(84.78%)	267(92.71%)	1	**1**	
**History of alcohol consumption**					
Yes	16(17.39%)	23(7.99%)	2.42(1.22,4.82)*	2.49(1.22,5.06)**	**0.01**
No	76(82.61%)	265(92.01%)	1	1	
**Perceived stress level**					
High level	33(35.87%)	103(35.76%)	1.04(0.61,1.63)	1.30(1.15,1.69)**	**0.03**
Low level	59(64.13%)	185(64.24%)	1	1	

**Keynote**: * statistically significant at bivariate analysis, ** statistically significant at multivariable analysis with *P*-value < 0.05

## Discussion

Abnormal uterine bleeding (AUB) is one of the most common gynecologic complaints among reproductive-age women. It is the commonest presenting symptom and major gynecological problem responsible for as many as one-third of all outpatient gynecologic visits [18]. This study has the potential to significantly contribute to improving women’s health in Southern Ethiopia and beyond. The identification of factors associated with AUB can guide healthcare planning and policy-making.

Therefore, this study aimed to assess the magnitude of AUB and its associated factors among reproductive-age women in Dilla University General Hospital. According to this study, the magnitude of abnormal uterine bleeding was 24.21% (95% CI, 20.14–28.79). This study coincided with previous studies from developing countries (8–30%) [4, 18] and Iran (27.2%) [12]. This finding was lower than studies done in Jimma (34.1%) [13], Debre Berhan (33.4%) [19], India (36%) [20], Iran (35.8%) [12], and Brazil (31.4%) [21]. This finding was also higher than that of a Chinese study (18.2%) [22]. The variations observed in the data could potentially be a result of the distinct methodologies implemented in the studies. However, other factors such as disparities in social, demographic, definitions of AUB, and cultural attributes, along with the level of access to health services, could also contribute to these differences.

A history of STIs was significantly associated with abnormal uterine bleeding. This finding is consistent with the study conducted in India [20] and Jimma [13]. This association might be explained by the fact that ascending infection causes uterine and Fallopian tube irritation, which leads to tubal dysfunction. It’s also possible that sexually transmitted infections (STIs) could lead to inflammation and infection in the reproductive system, disrupting the regular menstrual cycle and resulting in abnormal uterine bleeding (AUB).

The findings of this study revealed a significant association between anemia and menstrual irregularity. This is in line with studies conducted in Debre Berhan [17], and India [23]. In terms of stress, this finding revealed that a high level of perceived stress was associated with menstrual irregularity. A study conducted in China [24] and Debre Berhan [17, 19] supported this finding. This could be due to psychological stress, which could impair HPO-Axis coordination. Another possible reason may be stress affects the menstrual cycle. When stress levels are high, the HPA axis activity is disrupted. As a result, women who are under a lot of stress may have more irregular periods than those who have low-stress levels.

The association between excessive alcohol consumption and menstrual irregularity was found to be significant in this study. According to a study conducted in China [22], Serbia [25], and Debre Berhan [17], those who drink regularly are more likely to report heavy periods than never drinkers. The possible explanation may be women who drink alcohol have higher levels of testosterone, estrogen, and luteinizing hormone [26]. In turn, such hormonal imbalance may result in menstrual irregularity, implying biological plausibility for the association between alcohol consumption and abnormal uterine bleeding. However, more research is needed to fully understand this relationship.

This study’s limitation was the presence of self-reported symptoms of abnormal menstrual bleeding patterns in the questionnaire, which may not indicate a problem. Additionally, because the study was cross-sectional in nature, cause and effect were not determined. The study was conducted in a single hospital, which may limit the generalizability of the findings to the wider population of reproductive-age women in Southern Ethiopia or other regions.

### Conclusions and recommendations

The study revealed that the magnitude of AUB was 24.2% in the study setting and history of STI, history of anemia, history of alcohol consumption, and perceived stress level were found to be significantly associated with abnormal uterine bleeding. So local health planners, health institutions, health bureaus, and policymakers for routine screening and diagnosis of AUB for implementing appropriate strategies to decrease maternal morbidity and mortality due to abnormal uterine bleeding. Additional studies and interventions focusing on these risk factors are needed to enhance the overall health outcomes of those who experience abnormal uterine bleeding.

### Electronic supplementary material

Below is the link to the electronic supplementary material.

Supplementary Material 1

## Data Availability

All relevant data included in the manuscript are available from the corresponding author upon reasonable request.

## Abbreviations

AUB: Abnormal Uterine Bleeding
AOR: Adjusted Odd Ratio
BMI: Body Mass Index
CI: Confidence Interval
HPA: Hypothalamus Pituitary Adrenal
IUCD: Intra Uterine Contraceptive Device
STI: Sexual Transmitted Infections
PSS: Perceived Stress Scale

## References

[1] Melville C. Sexual and reproductive health at a glance. Wiley; 2015.

[2] Kaunitz AM, Levine D. Abnormal uterine bleeding in nonpregnant reproductive-age patients: evaluation and approach to diagnosis. UpToDateToDate; 2021.

[3] Sarala V, Gopalan U (2020). Clinical pattern and presentation of abnormal uterine bleeding. Int J Reprod Contracept Obstet Gynecol.

[4] Sun Y, Wang Y, Mao L, Wen J, Bai W. Prevalence of abnormal uterine bleeding according to new International Federation of Gynecology and Obstetrics classification in Chinese women of reproductive age: a cross-sectional study. Medicine. 2018;97(31). 10.1097/MD.0000000000011457 PMC6081150 30075511

[5] Wouk N, Helton M (2019). Abnormal uterine bleeding in premenopausal women. Am Family Phys.

[6] Rindfleisch K, Falleroni J, Schrager S (2015). Abnormal uterine bleeding in reproductive-aged women. J Clin Outcomes Manag.

[7] Whitaker L, Critchley HO (2016). Abnormal uterine bleeding. Best Pract Res Clin Obstet Gynecol.

[8] Choudhury SA, Nath P (2020). Abnormal uterine bleeding; its prevalence, causes and management in a tertiary care hospital. N Indian J OBGYN.

[9] Saheta A, Hariharan C, Sharma U (2014). Abnormal uterine bleeding. J Dent Med Sci.

[10] ÖZKARA A, ABNORMAL UTERINE BLEEDING:. A REVIEW. CURRENT APPROACHES IN GYNECOLOGY AND GYNECO-ONCOLOGY. 2022:133.

[11] Matthews ML (2015). Abnormal uterine bleeding in reproductive-aged women. Obstet Gynecol Clin.

[12] Kazemijaliseh H, Tehrani FR, Behboudi-Gandevani S, Khalili D, Hosseinpanah F, Azizi F. A Population-based study of the prevalence of abnormal uterine bleeding and its related factors among Iranian Reproductive-Age women: an updated data. Archives Iran Med (AIM). 2017;20(9). 29048917

[13] Gerema U, Kene K, Abera D, Adugna T, Nigussie M, Dereje D (2022). Abnormal uterine bleeding and associated factors among reproductive age women in Jimma town, Oromia Region, Southwest Ethiopia. Women’s Health.

[14] Jung E-K, Kim S-W, Ock S-M, Jung K-I, Song C-H (2018). Prevalence and related factors of irregular menstrual cycles in Korean women: the 5th Korean National Health and Nutrition Examination Survey (KNHANES-V, 2010–2012). J Psychosom Obstet Gynecol.

[15] Fraser IS, Langham S, Uhl-Hochgraeber K (2009). Health-related quality of life and economic burden of abnormal uterine bleeding. Expert Rev Obstet Gynecol.

[16] Cohen S. Perceived stress in a probability sample of the United States. 1988.

[17] Zeru AB, Gebeyaw ED, Ayele ET (2021). Magnitude and associated factors of menstrual irregularity among undergraduate students of Debre Berhan University, Ethiopia. Reproductive Health.

[18] Saheta A, Hariharan C, Sharma U (2014). Abnormal uterine bleeding. IOSR J Dent Med Sci.

[19] Mittiku YM, Mekonen H, Wogie G, Tizazu MA, Wake GE. Menstrual irregularity and its associated factors among college students in Ethiopia, 2021. Front Global Women’s Health. 2022;3. 10.3389/fgwh.2022.917643 PMC9445616 36081684

[20] Rathi BA, Chaudhari SC (2017). A clinical profile and factors associated with dysfunctional uterine bleeding at tertiary health care center. MedPulse Int J Gynaecol.

[21] Rezende GP, Yela Gomes DA, Benetti-Pinto CL (2023). Prevalence of abnormal uterine bleeding in Brazilian women: Association between self-perception and objective parameters. PLoS ONE.

[22] Ding C, Wang J, Cao Y, Pan Y, Lu X, Wang W (2019). Heavy menstrual bleeding among women aged 18–50 years living in Beijing, China: prevalence, risk factors, and impact on daily life. BMC Womens Health.

[23] Mohite R, Mohite V, Kumbhar S, Ganganahalli P. Common menstrual problems among Slum adolescent girls of Western Maharashtra, India. J Krishna Inst Med Sci (JKIMSU). 2013;2(1).

[24] Ansong E, Arhin SK, Cai Y, Xu X, Wu X (2019). Menstrual characteristics, disorders and associated risk factors among female international students in Zhejiang Province, China: a cross-sectional survey. BMC Womens Health.

[25] Ivanović R, Joksimović B, Čančar V, Marić H, Matović D, Lalović N (2024). Factors Associated with abnormal uterine bleeding in Perimenopausal Women. Clin Exp Obstet Gynecol.

[26] Erol A, Ho AMC, Winham SJ, Karpyak VM (2019). Sex hormones in alcohol consumption: a systematic review of evidence. Addict Biol.

