# Supplementary material for: Abnormal uterine bleeding and its associated factors among reproductive-age women who visit the gynecology ward in Dilla University General Hospital, Southern Ethiopia, 2022
Source: BMC Womens Health. 2024 May 9;24:281. doi: 10.1186/s12905-024-03128-6 (PMC11080245; doi:10.1186/s12905-024-03128-6)
Supplement: Supplementary file 1 — Supplementary Material 1 [file 12905_2024_3128_MOESM1_ESM.docx]

**English Versions Questionnaire**

**Part I: Socio-demographic characteristics of the study participants**

| Code | Variable | Response | Skip |
| --- | --- | --- | --- |
| 101 | Age | ---------------in years |  |
| 102 | Height | -------------- in Meter |  |
| 103 | Weight | ---------------in Kg |  |
| 104 | Residence | 0. Urban  1. Rural |  |
| 105 | Marital status | 1. No marriage history 2. marriage history |  |
| 106 | Educational level | 1. Can’t read and write 2. Can read and write 3. Primary school 4. Secondary school 5. College and above |  |
| 107 | Occupation status | 1. Housewife 2. Farmer 3. Daily Laborer 4. Merchant 5. Employee |  |
| 108 | Ethnicity | 0. Gedeo  1. Silit  2. Guraghe  3. Other/ specify----- |  |
| 109 | Religion | 0. Orthodox  1. Muslim  2. Protestant  3. Other/ specify |  |
| 110 | The monthly average income in ETB | ------------------- |  |

**Part II: - Lifestyle factors, reproductive history, and clinically diagnosis characteristics of the study participants**

| Code | Variable | Response | Remark/note |
| --- | --- | --- | --- |
| 111 | How many pregnancies do you experience? | 1. Nulliparous 2. Multiparous |  |
| 112 | Do you have a previous history of abortion? | 0. No  1. Yes |  |
| 113 | Do you have a previous history of IUCD use | 0. No  1. Yes |  |
| 114 | Do you have a previous history of hormonal contraceptive use | 0. No  1. Yes |  |
| 115 | Do you have a previous history of STI | 0. No  1. Yes |  |
| 116 | Do you have a previous history of Uterine cancer | 0. No  1. Yes |  |
| 117 | Do you have a previous history of Bleeding disorder | 0. No  1. Yes |  |
| 119 | Do you have a previous history of diagnosed anemia | 0. No  1. Yes |  |
| 120 | Do you have a history of cigarette smoking? | 0. No  1. Yes |  |
| 121 | If Yes to the above question, how often did you smoke? | 1. Never 2. Occasionally smoke 3. Regularly |  |
| 122 | Do you have a history of alcohol drinking? |  |  |
| 123 | If Yes to the above question, how often did you drink? | 1. Never 2. Occasionally Drinking 3. Regularly drinking |  |

Part III: Menstrual related questions

| Code | Variable | Response | Skip |
| --- | --- | --- | --- |
| 125 | Have you irregular uterine bleeding | 1. No 2. Yes |  |
| 126 | If **yes Q 125**, which type of irregular menstrual bleeding? | 1. Heavy periods 2. Metrorrhagia 3. Polymenorrhea 4. Oligomenorrhea 5. Amenorrhea 6. Inter-menstrual bleeding |  |

Part IV: Perceived stress scale

The questions in this scale ask you about your feelings and thoughts during the last month. In each case, you will be asked to indicate by circling how often you felt or thought a certain way

0 = Never 1 = Almost Never 2 = Sometimes 3 = Fairly Often 4 = Very Often

| In the last month, how often have you been upset because of something that happened unexpectedly? | 0 | 1 | 2 | 3 | 4 |
| --- | --- | --- | --- | --- | --- |
| In the last month, how often have you felt that you were unable to control the important things in your life? |  |  |  |  |  |
| In the last month, how often have you felt nervous and “stressed”? |  |  |  |  |  |
| In the last month, how often have you felt confident about your ability to handle your personal problems? |  |  |  |  |  |
| In the last month, how often have you felt that things were going your way? |  |  |  |  |  |
| In the last month, how often have you found that you could not cope with all the things that you had to do? |  |  |  |  |  |
| In the last month, how often have you been able to control irritations in your life? |  |  |  |  |  |
| In the last month, how often have you felt that you were on top of things? |  |  |  |  |  |
| In the last month, how often have you been angered because of things that were outside of your control? |  |  |  |  |  |
| In the last month, how often have you felt difficulties were piling up so high that you could not overcome them? |  |  |  |  |  |
